# Endometrial Adenocarcinoma in A 31-Year Old Woman: A Case Report

**DOI:** 10.22074/ijfs.2015.4249

**Published:** 2015-07-27

**Authors:** Firoozeh Ahmadi, Farnaz Akhbari, Zohreh Rashidi, Mandana Hemmat

**Affiliations:** 1 Department of Reproductive Imaging at Reproductive Biomedicine Research Center, Royan Institute for Reproductive Biomedicine, ACECR, Tehran, Iran; 2 Department of Endocrinology and Female Infertility at Reproductive Biomedicine Research Center, Royan Institute for Reproductive Biomedicine, ACECR, Tehran, Iran

**Keywords:** Endometrial Adenocarcinoma, Polycystic Ovarian Syndrome, Abnormal Uterine Bleeding, Infertility

## Abstract

Endometrial adenocarcinoma (EC) usually occurs after menopause, whereas in 2-14% of cases, it occurs in young patients (less than 40 years old) who may desire to keep their fertility. It is of importance to evaluate women for EC when they develop polycystic ovarian syndrome and abnormal uterine bleeding. Its treatment includes hysterectomy, bilateral salpingo-oo- phorectomy and pelvic lymphadenectomy and in some cases, radiation therapy. We report a case of EC in a 31-year-old woman who presented to Royan Institute. She complained about oligomenorrhea with a 10-year history of primary infertility.

## Introduction

Endometrial adenocarcinoma (EC) is the most common gynecologic malignancy in the United States, predominantly among postmenopausal women, at the average age of 59 years. The majority of cases are diagnosed when the carcinoma is confined to the uterus, leading to less than 1.5% of cancer deaths (1, 2).

Although 20-25% of EC are diagnosed before the menopause, 2-14% occur among younger women (less than 40), most of whom wish to preserve their fertility (3-6). This complication is more common in developed countries than the developing countries (7).

We report a case of a 31-year-old patient with an endometrial cancer diagnosed at stage II according to the International Federation of Gynecology and Obstetrics (FIGO), 2000 classification of endometrial cancer (8). This case study will provide a useful guide in diagnosis of EC for the sonographer.

The aim of study is to provide opportunity for sonographists to learn the broad spectrum of findings that may be seen at sonohysterography (SHG) in both benign and malignant processes to raise clinician’s awareness toward the appropriate diagnosis and treatment.

## Case Report

**Fig.1:**
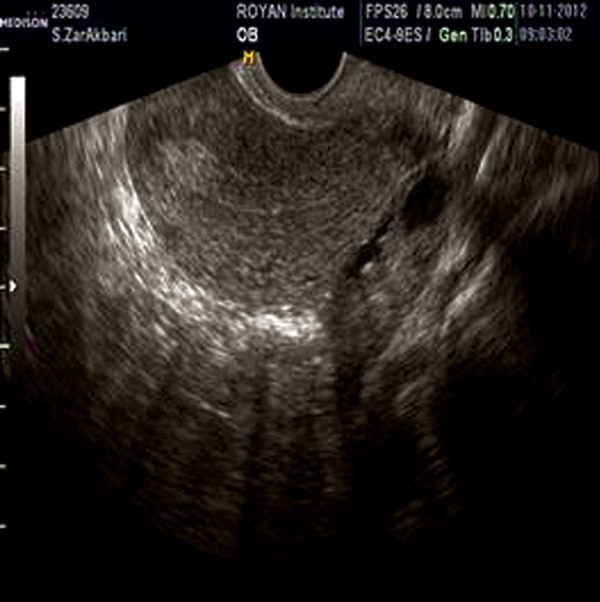
Sagital transvaginal sonogram showing anteverted uterus and endometrial layer of 12 mm.

**Fig.2:**
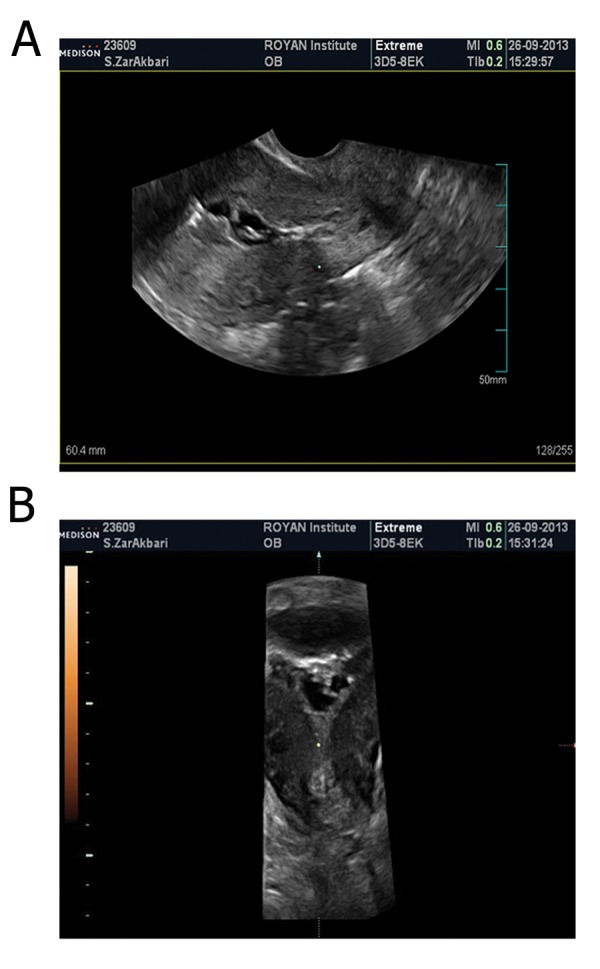
Sagital and coronal saline hysterosonogram showing several echogenic bands with irregular internal border.

A 31-year-old woman who was nulliparous and overweight [body mass index (BMI)=35.5] with a 10-year history of primary infertility presented to the Imaging Department of Royan Institute in 2012. She had a history of laparoscopy with ovarian cotter, septum and polyp resection by hysteroscopy (HSC) in 2010 at different infertility center. In addition, due to male factor infertility, she underwent ovarian stimulation in IVF cycle and ten embryos were obtained and frozen in 2010. Her chief complaint was oligomenorrhea. Since she was overweight and had abnormal uterine bleeding (AUB), transvaginal sonography (TVS) and SHG were done for patient. TVS showed thickened endometrium with smooth counter (Fig.1), while the result of three dimensional sonohysterography (3DSHG) revealed irregular endometrium and fibrotic bands which involved ½ of uterine cavity in fundus and body of the uterus, therefore, this finding proposed intrauterine adhesions (Fig.2A, B). Based on findings at TVS and SHG and consideration of irregular endometrium, she was then considered as a candidate for hysteroscopy operation and dilation and curettage (D&C). The hysteroscopic appearance of the endometrium consisting of multiple polypoid areas, indicated that patient was suspected of having hyperplasia or endometrial cancer, so direct biopsy was done and the specimen was then sent to pathological exam for further examination. The pathology report revealed endometrial adenocarcinoma (stage II). In order to determine the staging of disease, pelvic MRI was done. The result of pelvic MRI just showed endometrial involvement and other pelvic organs were normal, finally after completing oncologic evaluation, conservative management was not considered because she was patient decided to have the surgery to obtain her health promotion, so she underwent hysterectomy with bilateral salpingo-oophorectomy and pelvic lymphadenectomy. Regarding the fact that she had 10 embryos frozen two years ago, she could have a chance of surrogate pregnancy.

This is an interesting case because she was at reproductive age and adenocarcinoma is uncommon in this age group. Also obtained findings from transvaginal ultra sonography (TVUS) and SHG are not specific for adenocarcinoma.

## Discussion

The sonographic appearance of the normal endometrium highly depends on the age of the patient and the stage of menstrual cycle for woman of reproductive age.

A carcinoma generally originates from the epithelial cells. EC is a type of uterine cancer that involves the endometrium. Adenocarcinoma (adeno=gland) refers to a carcinoma featuring microscopic glandular-related tissue. Prior to the 1980s, EC was broadly characterized as a single disease. However, observations by Lauchlan (9), Hendrickson et al. (10) have led to the description of two distinct types based on histologic and molecular characteristics. Type I EC is commonly referred to endometrioid adenocarcinoma which includes 80-90% of all ECs. Type II EC, nonendometrioid tumors, encompasses the remaining 10- 20% of endometrial tumors (11). The etiology and survival of these two subtypes are vastly different.

The risk factors for EC which are related directly or indirectly to estrogen exposure including early menarche, late menopause, nullparity, poly cyctic ovarian syndrome (PCO), diabetes and obesity. Besides, adenomatous polyps, breast cancer and the use of estrogen therapy, are associated with higher incidence of endometrial cancer (6, 12-17). In our case, she had obesity, PCO, and adenomatous polyp, while she was considered nulliparous. History of estrogen use is more frequently seen in young patients. Also hormone-related disorders such as ovarian dysfunction, chronic anovulation, infertility, obesity and PCO detected in these patients (18). In this case, it should be mentioned that she had one stimulation cycle of assisted reproduction treatment (ART) in 2010, and subsequently, had 10 frozen embryos.

Physicians mostly prefer to perform endometrial biopsy if there is abnormal uterine bleeding which is considered as indication of early symptom of EC (18, 19). Although postmenopausal bleeding is a common sign of EC, recent studies have shown that only 4-5% of women with postmenopausal bleeding have endometrial cancer (1, 2).

SHG and TVUS provide a good predictive value for endometrial disease in patients with AUB (20). SHG which is instillation of sterile saline solution by means of a catheter was initially described by Nannini et al. (21). SHG produces better images, whereas TVUS provides more accurate measurement of the endometrial thickness allowing more clear evaluation of the heterogeneity. SHG has greater accuracy in the identification of focal lesions than in the diagnosis of diffuse lesions particularly more associated to malignant lesions. Adding 3D imaging to SHG can help getting optimal results because of allowing real time visualization of the cavity and more accurate assessing than conventional 2D. Other advantages are the ability to observe the coronal plane of the uterus and saving 3D volumes for later study, which leads to reduce the duration of examination and to cause less discomfort for the patients. In premenopausal patients, SHG is preferably performed during the early proliferative phase of the patient’s menstrual cycle, when the endometrium is a very thin tissue. Although there is no limitation for normal premenopausal endometrial thickness, the endometrium should be uniform in thickness, homogeneous in echotexture, and not to be displaced by any submucosal, myometrial abnormality (22).

In postmenopausal patients, the normal atrophic endometrium should measure be less than 4 mm in double-layer thickness as seen at TVUS and less than 2.5 mm in single-layer thickness as seen at SHG. In addition, the atrophic endometrium should be smooth and uniform in echotexture and not to be displaced by any submucosal, myometrial abnormalities (19, 23).

The most common appearance of EC at TVUS is nonspecific thickening of the endometrium. Even at SHG, endometrial cancer can be difficult to distinguish from endometrial hyperplasia and polyps. This diagnosis should be suspected when the single layer of the endometrium is thicker than 8 mm, irregular, broad based, or poorly marginated or when the endometrial-myometrial interface is disrupted. One of interest finding in our case is that she had smooth endometrial-myometrial interface. Endometrial thickness measurements often overlap in benign and malignant conditions (24).

At SHG, EC is typically a more diffuse process, while early cases can appear as a polypoid mass (19). An intact subendometrium shows localized disease, whereas extension of heterogeneity and increased echogenicity in the myometrium propose advanced invasive endometrial carcinoma (25). Sonographic findings related to EC in our patient included a thickened, heterogeneous endometrium.

Final diagnosis achieved through HSC finding and pathology. Advantages of HSC in the evaluation of abnormal bleeding or abnormal lesion are notable and the ability to see lesions and to evaluate endometrial cavity is precious. The panoramic HSC, especially with directed biopsy is superior to D&C in patient with abnormal findings (22).

## Conclusion

SHG make obvious differentiation between focal and diffuse endometrial lesions, so it becomes reliable test in the imaging evaluation of dysfunctional uterine bleeding and postmenopausal bleeding. It is essential for the radiologist to be familiar with the broad spectrum of findings that may be seen at SHG in both benign and malignant processes in order to direct the clinician toward the appropriate means of diagnostic biopsy or surgery.
